# Anti-Suicidal Effects of Lithium, Ketamine, and Clozapine—A 10-Year Systematic Review

**DOI:** 10.3390/ph18050742

**Published:** 2025-05-18

**Authors:** Przemyslaw M. Waszak, Jan Opalko, Natalia Olszańska, Paweł Zagożdżon

**Affiliations:** 1 Department of Hygiene & Epidemiology, Medical University of Gdansk, ul. Dębinki 7, 80-211 Gdańsk, Poland; 2 Faculty of Medicine, Medical University of Gdansk, 80-309 Gdansk, Poland; 3 Independent Public Healthcare Institution, Ministry of Interior and Administration, 02-507 Gdańsk, Poland

**Keywords:** suicide, suicidality, suicidal ideation, lithium, clozapine, ketamine, systematic review

## Abstract

**Background/Objectives:** Suicide is a complex issue resulting in approximately 700,000 deaths annually. Individuals with mood disorders or schizophrenia are at an increased risk. Pharmacological interventions, such as lithium, clozapine, and ketamine, show promise in reducing suicidality. **Methods:** A systematic search was conducted across Google Scholar, Scopus, and PubMed to identify studies evaluating the effects of lithium, clozapine, and ketamine on suicidality. Peer-reviewed articles published between 2014 and 2024 that focused on adult populations were included. After screening 1297 records, 49 studies met the eligibility criteria: 14 on lithium, 23 on ketamine, and 12 on clozapine. **Results:** Multiple studies highlight lithium’s significant anti-suicidal effects in patients with bipolar disorder, showing superior suicide risk reduction compared to valproate and other mood stabilizers. Ketamine has been shown to rapidly reduce suicidal ideation, with effects observable within hours and lasting up to a week. While most studies support its short-term efficacy, findings regarding its long-term benefits and the impact of repeated dosing remain inconsistent. Clozapine has consistently demonstrated a reduction in suicide risk for individuals with schizophrenia. Large-scale cohort studies report a significant decrease in suicide attempts and mortality when compared to other antipsychotics. **Conclusions:** Lithium, ketamine, and clozapine were proven to be effective in reducing suicidality. However, limited data, adherence challenges, and methodological differences across studies highlight the need for more robust, large-scale experimental research. Effective suicide prevention is an extremely complex topic and also requires consideration of healthcare and social system factors.

## 1. Introduction

Suicide is a complex phenomenon that arises from the interplay of biological, psychological, and social factors. According to the World Health Organization, approximately 700,000 people die by suicide each year worldwide, with many more attempting suicide or experiencing serious suicidal thoughts [1,2]. Suicide is especially prevalent among individuals with mood disorders, such as major depressive disorder (MDD) and bipolar disorder (BD), as well as those diagnosed with schizophrenia spectrum disorders [3]. As a result, ensuring broad access to mental health care and providing effective treatment are essential pillars of suicide prevention [2].

While effective treatment of mental disorders is generally believed to reduce suicidal behavior indirectly, only a few medications have demonstrated a direct anti-suicidal effect. Among pharmacological interventions, three agents—lithium, clozapine, and ketamine—have consistently emerged as promising treatments with significant anti-suicidal properties [4,5].

### 1.1. Lithium

Lithium salts have been widely used in the treatment of mood disorders for over 70 years. They are effective in reducing the frequency, severity, and duration of mood episode relapses, thereby improving long-term stability and quality of life in patients. Today, lithium remains one of the first-line medications for preventing recurrences in both type I and type II bipolar disorder.

Although concerns have been raised about its safety, particularly with respect to hypothyroidism, electrocardiographic changes, and potential nephrotoxicity, lithium is generally considered safe when managed with appropriate medical supervision. Maternal use of lithium during the first trimester has been associated with an increased risk of cardiac malformations, including Ebstein’s anomaly.

A key aspect of lithium therapy is that dosing must be individualized based on serum levels; therefore, regular blood tests are required throughout treatment [6,7]. Despite decades of use, the exact mechanism of lithium’s action remains incompletely understood. It is known to exert complex effects on both intracellular and extracellular signaling pathways [8]. Notably, lithium appears to reduce aggression and impulsivity—traits that may contribute to its unique anti-suicidal properties [9].

### 1.2. Ketamine

Ketamine is an N-methyl-D-aspartate (NMDA) receptor antagonist that has long been recognized as a safe anesthetic agent with notable analgesic properties [10]. Esketamine, which contains only the (S)-enantiomer of ketamine, is primarily administered as a nasal spray [11]. In contrast, arketamine—the (R)-enantiomer—is less potent as both an NMDA receptor antagonist and an anesthetic, and it has not been approved for clinical use [12].

Beyond anesthesia, ketamine’s therapeutic applications have expanded to include pain management, procedural sedation, and, increasingly, psychiatric treatment [13]. Growing evidence supports its rapid antidepressant effects, even in patients with treatment-resistant depression, following a single subanesthetic dose [14]. This rapid onset of action sets ketamine apart from conventional antidepressants, which typically require weeks to achieve noticeable effects [15].

The antidepressant mechanisms of ketamine are thought to be multifaceted. These include NMDA receptor inhibition on γ-aminobutyric acid (GABA) interneurons, leading to disinhibition of glutamatergic transmission, and subsequent activation of α-amino-3-hydroxy-5-methyl-4-isoxazolepropionic acid (AMPA) receptors through increased glutamate release [16]. Esketamine has been approved by both the U.S. Food and Drug Administration (FDA) and the European Medicines Agency (EMA) for the treatment of treatment-resistant major depressive disorder [17].

Interestingly, lithium and ketamine share overlapping neurobiological pathways involved in neuroplasticity and antidepressant effects. Ketamine rapidly enhances AMPA receptor activity and increases brain-derived neurotrophic factor (BDNF), which activates the mTOR signaling pathway and inhibits glycogen synthase kinase-3 beta (GSK-3β). Lithium also activates the mTOR pathway and inhibits GSK-3β, contributing to synaptic growth and plasticity. Preclinical studies suggest that lithium may potentiate ketamine’s antidepressant effects in models resistant to ketamine alone [18]. However, clinical evidence remains mixed. While lithium may not enhance ketamine’s efficacy in all patients, it could offer benefits in specific subgroups, particularly those with bipolar disorder [19,20]. Nonetheless, combining lithium and ketamine should be approached with caution due to the potential for additive adverse reactions [19].

### 1.3. Clozapine

Clozapine is the only antipsychotic approved by the FDA specifically for patients with treatment-resistant schizophrenia, defined as persistent symptoms despite adequate trials of at least two other antipsychotic medications [21]. It is widely regarded as the gold-standard treatment in this population due to its superior efficacy [21,22]. Long-term studies have demonstrated that clozapine reduces overall mortality in individuals with schizophrenia, largely due to decreased suicide rates and improved symptom control, which may help prevent medical complications and psychosocial deterioration [21]. Despite its clear benefits, clozapine is associated with a complex and potentially serious side effect profile. These include agranulocytosis (severe neutropenia), myocarditis, metabolic syndrome, seizures, and other adverse effects [23,24]. As a result, rigorous hematological monitoring—specifically regular assessments of white blood cell and neutrophil counts—is mandatory. Broader clinical surveillance is also required to detect and manage other complications early. Due to these challenges, clozapine remains underused or is introduced late in the treatment course, which may negatively affect patient outcomes [23].

Importantly, clozapine is the only antipsychotic approved by the FDA to reduce recurrent suicidal behavior in individuals with schizophrenia [25]. The landmark InterSePT trial—a two-year randomized study—found that clozapine reduced suicide attempts by 24–26% compared to olanzapine in patients with schizophrenia or schizoaffective disorder who were at high risk of suicide. Significant outcomes included fewer suicide attempts, hospitalizations, and rescue interventions [26]. In comparison to other antipsychotics, clozapine is more effective in alleviating core schizophrenia symptoms as well as comorbid depressive symptoms, both of which may contribute to its anti-suicidal effects [25]. Some studies also suggest that clozapine’s positive effects on sleep may mediate its impact on suicidality [27]. Basic research has revealed that clozapine acts through complex, multifactorial mechanisms, with no single pathway fully explaining its efficacy [27,28]. Its anti-suicidal properties may involve several neurobiological systems. These include modulation of the norepinephrine system, high affinity for 5-HT2A receptors, and influence on NMDA receptor expression [25,28]. Given that suicide is often conceptualized as self-directed aggression, clozapine’s anti-aggressive effects may also play a role. Furthermore, clozapine has been shown to reduce substance use—a known and significant risk factor for suicide [25].

This review aims to synthesize the literature from the past decade, focusing on the efficacy of lithium, clozapine, and ketamine in reducing suicidality. We explored the last 10 years of research, aiming to provide a comprehensive overview of the current evidence base, highlight potential mechanisms of action, and discuss both limitations and implications for clinical practice and future research.

## 2. Methods

### 2.1. Search Strategy

Databases Google Scholar, Scopus, and PubMed were searched using a query designed to capture articles investigating the effects of lithium, clozapine, ketamine, and esketamine on suicidality (suicidal ideation, suicidal behavior, or suicide risk). The main search query combined Mesh terms and Title/Abstract keywords:

*((“Lithium” [Mesh] OR lithium [Title/Abstract]) OR (“Clozapine” [Mesh] OR clozapine [Title/Abstract]) OR (“Ketamine” [Mesh] OR ketamine [Title/Abstract]) OR (“Ketamine, (S)-” [Supplementary Concept] OR esketamine [Title/Abstract]))*

*AND*

*(“Suicide” [Mesh] OR “Suicidal Ideation” [Mesh] OR suicide [Title/Abstract] OR suicidal [Title/Abstract] OR suicidality [Title/Abstract] OR “anti-suicidal effect” [Title/Abstract] OR “antisuicidal effect” [Title/Abstract])*

Only articles published in the last 10 years (2014–2024) were included.

### 2.2. Inclusion and Exclusion Criteria

Studies were included if they met the following criteria: (1) peer-reviewed publications in English; (2) original research articles; and (3) primary focus on the effects of lithium, clozapine, or ketamine on suicide risk, suicidal behavior, and/or suicidal ideation. Only studies involving adult populations (aged 18 years or older) were considered.

Exclusion criteria were as follows: case reports, editorials, letters to the editor, commentaries, and conference abstracts that did not contain original data. Studies were also excluded if they did not explicitly assess or report on outcomes related to suicidal ideation, suicide attempts, or overall suicidality. Research focusing on pediatric populations was not included in this review.

### 2.3. Data Extraction and Synthesis

Three authors (J.O., N.O., P.W.) independently screened the results of the systematic search. Each title and, when available, abstract were reviewed to determine relevance based on the predefined inclusion and exclusion criteria. Any discrepancies were resolved through discussion and consensus. Additionally, the reference lists of the included studies were screened to identify further relevant publications.

**Figure 1:**
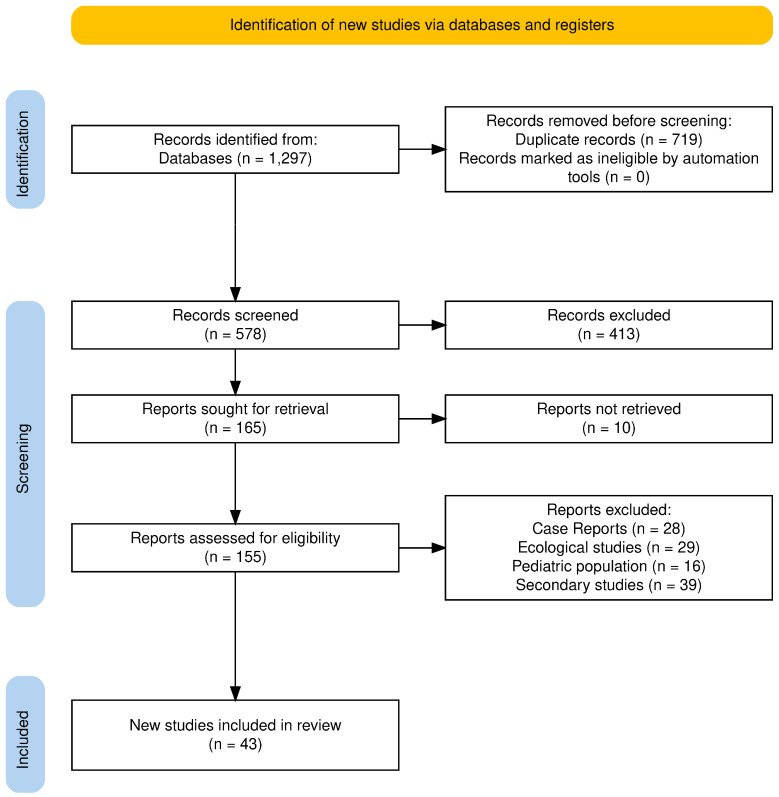
PRISMA 2020 flow diagram.

The initial search yielded 1297 records. After the removal of 719 duplicates, 578 unique records remained for screening. Of these, 413 were excluded based on title and abstract screening, leaving 165 full-text reports for retrieval. Ten reports could not be accessed, resulting in 155 studies assessed for eligibility. Following full-text review, 106 studies were excluded for reasons such as them being case reports, ecological or autopsy studies, or otherwise not meeting the inclusion criteria. Ultimately, 43 unique studies were included in the final analysis: 13 on lithium, 23 on ketamine, and 7 on clozapine (Figure 1).

All studies reporting suicidal outcomes—such as suicidal ideation, suicide attempts, or completed suicide—were considered eligible for analysis. Data were extracted on key variables, including study design, sample size (including control groups, if applicable), inclusion and exclusion criteria, duration of follow-up, medication dosage (when reported), and primary study outcomes. All numerical results, regardless of effect size, were included.

Risk of bias was assessed collectively by all authors, with consideration given to factors such as study design, sample size, heterogeneity of findings, reported effect sizes, and methodological quality as described in each publication.

The extracted data were summarized and synthesized in Table 1, Table 2 and Table 3, corresponding to lithium, ketamine, and clozapine. No additional data visualization tools were employed. The preparation of this literature review was conducted in accordance with the PRISMA (Preferred Reporting Items for Systematic Reviews and Meta-Analyses) guidelines [29].

## 3. Results

### 3.1. Lithium and Suicidality

#### 3.1.1. Bipolar Patients

Individuals with BD face a markedly elevated risk of suicide, with estimates suggesting a 20- to 30-fold increase in suicide mortality compared to the general population [70]. As lithium is primarily prescribed in the treatment of BD, this patient group is particularly relevant for examining its anti-suicidal effects. In this review, six studies focusing specifically on BD populations were identified (Table 1).

A large population-based study conducted in Sweden between 2005 and 2013, involving 51,535 patients, found that lithium treatment significantly reduced the incidence of suicide-related events by 14% compared to periods without treatment. In contrast, valproate showed no protective effect [40]. The authors estimated that 12% of suicide-related events could have been prevented if patients had consistently adhered to lithium therapy, emphasizing the need for prioritizing lithium in treatment guidelines and ensuring proper patient support and monitoring [40].

Another long-term observational study from Hong Kong assessed all-cause and suicide-specific mortality in BD patients treated over a 16-year period with mood stabilizers such as lithium, valproate, quetiapine, and olanzapine [39]. The findings revealed that olanzapine and risperidone were associated with significantly higher all-cause mortality compared to lithium. Lithium and valproate had comparable mortality risks, reinforcing lithium’s relative safety and potential benefits in minimizing premature mortality [39].

A Taiwanese cohort study followed 25,787 patients with bipolar disorder over 16 years, comparing the impact of various mood stabilizers (lithium, valproate, lamotrigine, and carbamazepine) [37]. The study concluded that mood stabilizers—particularly lithium—were significantly associated with reduced risks of all-cause mortality, suicide, and natural death, with lithium demonstrating the lowest mortality risk among the agents studied [37].

Elena Toffol et al. analyzed 826 patients from a Finnish nationwide registry, focusing on high-risk BD individuals [35]. The study demonstrated that lithium was significantly associated with reduced suicide mortality and a 49% decrease in all-cause mortality. Conversely, treatment with antidepressants, valproic acid, and benzodiazepines was linked to increased risk of suicide attempts and mortality. However, the study’s limitations include non-standardized treatment protocols and the absence of structured diagnostic interviews [35].

A randomized controlled trial explored the anti-suicidal effects of lithium compared to valproate in high-risk bipolar patients, with a specific focus on age-related outcomes [30]. It found that patients aged 42 years or older on lithium have a significantly lower risk of suicidal behavior compared to patients > 42 years old on valproate or younger patients on either medication. The findings indicate that treatment with lithium may reduce suicide risk in bipolar patients > 42 years old, highlighting the importance of age-dependent differences in response to lithium. Nevertheless, the relatively small sample size (94 participants) may limit the generalizability of the findings [30].

#### 3.1.2. Anti-Suicidal Effect Studied in U.S. Veterans

Several studies have explored the potential anti-suicidal properties of lithium among U.S. veteran populations, a group characterized by high rates of mental health disorders and elevated suicide risk.

A small cohort study investigated the use of the Veterans Crisis Line and hospitalization rates among veterans treated with lithium [38]. The results indicated a statistically significant reduction in hospitalizations for both suicide attempts and suicidal ideation after six months of lithium treatment. Specifically, hospitalization for suicide attempts dropped from 4.1% to 0%, and admissions for suicidal ideation decreased from 13.3% to 1.0%. However, due to the limited sample size, the authors advised that these findings should be interpreted as hypothesis-generating rather than conclusive [38].

Eric Smith and colleagues conducted three studies examining lithium’s impact on mortality in veterans. Two of these studies analyzed similar cohorts, comparing lithium and valproate in terms of general and suicide-specific mortality outcomes [38,42]. One study reported that lithium was associated with a reduction in non-suicide mortality during active treatment compared to valproate [38]. The other found no significant difference in suicide mortality between lithium and valproate during treatment. However, both studies noted that lithium discontinuation was linked to a heightened risk of suicide within 180 days, suggesting that while lithium may offer protective effects during treatment, abrupt discontinuation can increase vulnerability—particularly in high-risk individuals [42].

A third study by Smith and colleagues examined veterans both with and without bipolar disorder, using high-dimensional propensity score matching to explore the association between lithium and suicide risk [41]. Among veterans with bipolar disorder, lithium initiation was linked to an increased one-year suicide risk, largely driven by elevated risk following discontinuation. In contrast, among those without bipolar disorder, lithium was associated with a significantly reduced suicide risk, underscoring the importance of diagnosis-specific effects and the consequences of treatment interruption [41].

Two studies were published based on randomized, placebo-controlled clinical trials. The authors chose different methodologies: intent-to-treat [32] and per-protocol [36] approaches. The two studies evaluate lithium’s potential in reducing suicidality among U.S. veterans with bipolar disorder or major depressive disorder, offering complementary insights through different techniques.

In the per-protocol study [36], patients receiving lithium had a lower 12-month suicidality risk (18.8%) compared to those receiving placebo (24.3%), yielding a risk ratio of 0.78. However, the results were inconclusive due to low adherence rates (only 17%), emphasizing the crucial role of treatment compliance in achieving benefit [36].

Conversely, the intention-to-treat trial [32] found no significant difference in repeat suicide-related events between the lithium (25.5%) and placebo (23.5%) groups. The hazard ratio was 1.10, indicating no clear advantage for lithium when added to standard care. Notably, discontinuation of any medication, regardless of group, was associated with a heightened risk of suicide-related outcomes [32]. Together, these studies underscore the potential of lithium as a protective agent, particularly when adherence is maintained. However, they also highlight the challenges in real-world implementation, especially in achieving consistent medication adherence and mitigating risks associated with treatment discontinuation.

#### 3.1.3. Other Populations

Ole Brus et al. examined the impact of lithium on suicide and readmission risks in patients with unipolar depression who had undergone electroconvulsive therapy (ECT) [34]. The study found that lithium significantly reduced both the risk of suicide (*p* = 0.014) and the risk of readmission (HR 0.84). The number needed to treat (NNT) to prevent one readmission was 16. These findings support the Swedish guidelines, which advocate for lithium use as a prophylactic measure following ECT. This suggests that lithium could play a more prominent role in the post-ECT management of these patients [34]. However, the study’s findings may not be generalizable to broader populations due to the specific cohort and treatment settings involved [34].

The final study identified was a randomized clinical trial [31] which involved only 56 patients, far below the target of over 200, leading to a statistically underpowered study. The results indicated that lithium, when added to usual care, did not significantly reduce deliberate self-harm after 12 months. This lack of effect is likely attributable to the insufficient sample size, which undermined the statistical power of the study.

Lithium shows strong and consistent evidence for long-term suicide prevention in bipolar disorder, outperforming other mood stabilizers like valproate and lamotrigine. However, high discontinuation and low adherence rates, ranging from 76.5% to as low as 17%, pose significant clinical challenges. These issues are linked to both the treatment regimen and the nature of bipolar disorder itself. Additionally, reduced suicide rates may be partly attributed to increased healthcare contact during lithium monitoring, suggesting a role of social factors beyond the drug’s direct effects.

Lithium levels in some of the studies were consistent between 0.6 and 0.8 mEq/dL. However, in as many as eight studies, they were not reported at all. The studies published between 2014 and 2024 also analyzed quite diverse populations (veterans, BD patients, European countries, Asian countries, the US). Quite a large group size (thousands of participants) was gathered by registry-based cohort-type studies. However, randomized controlled trials (RCTs) have recruited much smaller numbers of participants. Only two RCTs had about 500 patients, and the other two were underpowered and involved fewer than 100 patients. This rather high heterogeneity of the results makes it difficult to formulate more general conclusions.

### 3.2. Ketamine and Suicidality

We found 23 studies published between 2014 and 2024 that assessed ketamine’s anti-suicidal effects (Table 2). Out of these, twelve required subjects to have MDD, one required subjects to have bipolar disorder, one required subjects to have borderline personality disorder, and five did not require a specific psychiatric disorder as an inclusion criterion. The majority administered an intravenous (IV) 0.5 mg/kg dose of ketamine. Two studies assessed a 0.2 mg/kg dose, and another compared three doses of ketamine: 0.1, 0.5, and 1.0 mg/kg. Two studies administered a fixed dose of intranasal (IN) ketamine. Ten studies used a 0.02–0.05 mg/kg midazolam dose as a control, and the remaining nine used a saline solution.

Six studies assessed ketamine’s anti-suicidal effects in patients with major depressive disorder using saline as a control. Zolghadriha et al. and Domany et al. noted a rapid and significant reduction in suicidal ideation after the administration of a single IV ketamine infusion, with effects observed by 60 min and 90 min post-treatment, respectively [50,59]. An acute anti-suicidal effect of ketamine was also reported by Burger et al. Hu et al. investigated the impact of a single ketamine infusion adjunct to newly initiated antidepressant therapy [61,62]. Ketamine augmentation of escitalopram significantly lowered suicidal ideation between 2 h and 72 h post-infusion. A study by Ahmed et al. gave patients two infusions of 0.5 mg/kg ketamine 7 days apart and noted a significant reduction in suicide ideation one week after the second infusion [5]. However, contradictory findings have been observed. Ionescu et al. reported no significant advantage between repeated doses of ketamine in reducing suicidal ideation [56]. The contrasting results may be due to a couple of reasons. Most importantly, the study group had a greater level of chronicity and treatment resistance, with most patients failing five or more antidepressant trials, nearly 50% not responding to ECT treatment, and the current MDD episode being on average of 9.6 years long.

Six studies examined the effect of intravenous ketamine on suicidal ideation in patients with MDD using midazolam as a control. Grunebaum et al. found that a single ketamine infusion was associated with a significantly greater reduction in suicidal thoughts by 24 h post-treatment as assessed by the clinician-rated scale for suicidal ideation (SSI) [43,48]. This improvement was maintained during the 6-week follow-up; however, during this period medication changes to co-administered antidepressants were permissible. These findings are supported by Sinyor et al., who administered s ketamine infusions over 12 days [52]. The study found that ketamine infusions achieved a greater and longer-lasting (up to 42 days) reduction in suicidal ideation compared to the midazolam control. Both studies used ketamine as an adjunctive therapy and did not require subjects to have treatment-resistant depression. Five studies assessed ketamine’s anti-suicidal efficacy in treatment-resistant depression using midazolam as an active control. Phillips et al. found lower suicide item Montgomery–Åsberg Depression Rating Scale (MARDS-SI) scores 2 h and 7 days after a single ketamine infusion, and scores did not differ at 24 h [45]. Further reductions in MARDS-SI were seen during the administration of six open-label ketamine infusions over the course of two weeks, which ceased once ketamine infusions were reduced to once weekly. These weekly infusions were sufficient to sustain reductions in suicidal ideation. Both Price et al. and TP Su et al. observed a significant decrease in suicidal cognition 24 h after the administration of a single 0.5 mg/kg ketamine infusion [53,54]. In addition, TP Su et al. observed significant anti-suicidal effects of ketamine up to 5 days [53]. Similarly to Ionescu et al., TP-Su et al. noted that individuals whose current depressive episode lasted >24 months or who failed >4 antidepressant treatments did not benefit from ketamine’s anti-suicidal effects [51,53,56]. Feeney et al. found that among individuals with clinically significant suicidal ideation, those who had received ketamine had a significant reduction in suicidal ideation at 30 days post-infusion in comparison to those who had received midazolam [58]. However, a weakening effect was observed as early as day 3 post-infusion.

Grunebaum et al. evaluated the effect of a single 0.5 mg/kg infusion of ketamine on patients with bipolar depression and suicidal thoughts [43]. At day 1 post-infusion, a greater decrease in suicidal ideation was observed after the ketamine infusion than after midazolam. The difference was not statistically significant, which may be explained by the small sample size of 16 patients.

Ketamine’s anti-suicidal effect was also examined in patients with bipolar personality disorder and current suicidal ideation. However, Fineberg et al. found no significant decrease in suicidal ideation [44].

Three studies assessed the effects of intravenous ketamine in patients without a specific psychiatric diagnosis as an inclusion criterion. Murrough et al. and Abbar et al. investigated the efficacy of ketamine in patients with suicidal ideation and varying comorbid mental disorders [46,57]. Abbar et al. investigated the efficacy of two ketamine infusions administered 24 h apart vs. a saline placebo [46], whereas in the study conducted by Murrough et al., patients received a single IV infusion of ketamine or midazolam as a control [57]. It is worth noting that the exclusion criteria in both studies included psychotic disorders and substance dependance. Murrough et al. also excluded patients with a current intent to make a suicide attempt [57]. Abbar et al. found that a greater percentage of patients receiving ketamine achieved full remission of suicidal ideation 2 h after the first infusion and 72 h following the second infusion compared to the placebo group (43.8% vs. 7.3% and 63.0% vs. 31.6%, respectively) [46]. This effect was observed up to 6 weeks post-infusion; however, it was no longer statistically significant at 6 weeks. In Murrough et al.’s study, ketamine had a significant effect on suicidal ideation 48 h post-treatment, although this was not observed at 24 h [57].

Fan et al. found a significant decrease in suicidal ideation on day 1 and day 3 post-infusion of ketamine in patients with a new cancer diagnosis [55].

We found four studies assessing the anti-suicidal effects of esketamine. Three of which compared an 84 mg esketamine nasal spray to a placebo control in patients with mild depressive disorder and active suicidal ideation. All three studies repeated intakes of intranasal esketamine over a four-week period. One of these was a randomized controlled trial (RCT) conducted by Canuso et al. which found no differences between treatment groups at the end of the double-blind treatment or at post-treatment follow-up; however, a significant improvement on the MARDS suicidal item was observed 4 h post first dose [47]. Both Fu et al. and Ionescu et al. found no significant difference in suicidal ideation 24 h after first dosage [49,56]. Zeng et al. assessed esketamine as an ECT anesthetic in the treatment of MDD characterized by an inadequate response to at least two antidepressant therapies [60]. ECT was conducted three times per week, totaling eight sessions. Propofol was used as the control. The trial found no significant differences in anti-suicidal effects between the two groups.

Two studies analyzed the anti-suicidal effects of intranasal ketamine. Domany et al. administered a 40 mg IN dose of ketamine to patients requiring hospitalization due to suicide risk in an emergency department setting [59]. A significant reduction in suicidal ideation was observed in 4 h as measured by MARDS-SI; however, it was not confirmed by the self-reported BSS scale. Similar findings were seen by Jones et al. in patients with unipolar and bipolar depression with or without concomitant alcohol abuse [63]. Jones et al. found no significant change in SSI scores compared to a placebo; however, a significant improvement was seen in MARDS-SI [63].

Several meta-analyses have reported rapid and significant reductions in suicidal thoughts after the administration of a single ketamine infusion, with effects being observed within 2 h post-infusion [71]. The anti-suicidal duration of a single ketamine dose varies among studies between 24 h and 7 days post-infusion [71]. Contrasting results concerning repeated ketamine doses have been described, with Feng et al. observing no profit in the duration and efficacy of anti-suicidal ideation and Shen et al. revealing an additional benefit into the remission of suicidal behavior [72,73]. This calls for additional research in order to observe long-term effects and establish treatment protocols. The rapid anti-suicidal effect of ketamine may be implemented into the management of acute suicidality in an emergency setting. Ketamine could potentially reduce suicide mortality rates; however, further studies assessing its effect on actual suicidal behavior are required.

A significant convenience in comparing results is the consistency of ketamine dosage across the studies analyzed (mostly 0.5 mg/kg by infusion), but there was also some dose discrepancy. Most of the studies were RCTs, which reduces the risk of bias in research studies. However, the number of participants receiving active treatment was generally less than 100, indicating a rather underpowered study population. Only two RCTs had slightly more than 100 participants in the actively treated group, which is also hard to consider as groundbreaking.

However, ketamine demonstrated rapid but short-lasting anti-suicidal effects, making it a promising option for acute suicidal crises, particularly in depressive disorders where no other drug has shown such immediate impact. Unlike traditional antidepressants, which take weeks to become effective and lack proven anti-suicidal properties, ketamine reduces suicidal ideation within hours. However, its benefits are transient, and long-term efficacy remains uncertain. Further large-scale, real-world studies are needed before ketamine can be widely recommended in treatment guidelines.

### 3.3. Clozapine and Suicidality

The findings from studies published 2014–2024 consistently demonstrate that clozapine significantly reduces the risk of suicide and suicide attempts among patients with schizophrenia (Table 3). Multiple large-scale cohort studies, including those by Weitoft, van der Zalm, and Taipale, found that clozapine use was associated with a substantial reduction in suicide risk compared to other antipsychotics, with hazard ratios ranging from 0.21 to 0.66 [64,65,66,67].

Weitoft et al. examined 26,046 schizophrenia patients in Sweden between 2006 and 2009. The study found that clozapine users had a 55% lower risk of suicide (OR = 0.45; 95% CI: 0.20–0.98) and a 56% lower risk of attempted suicide (OR = 0.44; 95% CI: 0.28–0.70) compared to those using haloperidol [64].

Taipale et al. conducted a large-scale study in Finland (*n* = 61,889) and Sweden (*n* = 29,823) to compare the effects of different antipsychotics on suicide risk. Clozapine use was associated with a significant reduction in suicide risk, with hazard ratios (HRs) of 0.64 (95% CI: 0.49–0.84) in Finland and 0.66 (95% CI: 0.43–0.99) in Sweden. No other antipsychotic showed a similar protective effect. Conversely, benzodiazepines and Z-drugs were linked to an increased risk, with HRs ranging from 1.29 to 1.62 [66]

Van der Zalm et al. analyzed mortality among 22,110 Danish patients with first-time nonaffective psychotic disorders and a broader cohort of 50,881 individuals with prior diagnoses. Clozapine users had a lower suicide risk compared to those using other antipsychotics, with an adjusted HR of 1.76 (95% CI: 0.72–4.32) in the incidence cohort and 2.20 (95% CI: 1.35–3.59) in the prevalence cohort [65]. Interestingly, patients who discontinued clozapine within the first year were at significantly higher risk of suicide (HR = 0.65; 95% CI: 0.46–0.91), suggesting that cessation marks a high-risk period [65].

The protective effect was further supported by a study from Taiwan, which highlighted a dose-dependent reduction in suicide risk, where every 10-day increase in clozapine use led to a further decrease in risk [25]. Chen et al. (2024) conducted a nationwide cohort study in Taiwan using data from the National Health Insurance Research Database (2001–2019) to assess the impact of clozapine on all-cause, natural, and suicide mortality in patients with schizophrenia [25]. The study included 43,025 schizophrenia inpatients, of which 5800 received clozapine. Patients using clozapine had a 41% lower risk of all-cause mortality (HR = 0.59, 95% CI: 0.53–0.66). Suicide mortality risk was also reduced by 60% (HR = 0.40, 95% CI: 0.26–0.61) [25].

A small, single-center study by Gürcan et al. found that clozapine treatment significantly reduced suicide attempts in schizophrenia patients with a history of suicidality [69]. The study included 122 outpatients diagnosed with schizophrenia (*Diagnostic and Statistical Manual of Mental Disorders*; DSM-IV criteria) and assessed their suicide attempt rates before and after clozapine treatment. Prior to clozapine, 39.3% of patients had attempted suicide, whereas after treatment, this number dropped to just 7.4%. Notably, among 39 patients (32%) who had attempted suicide before starting clozapine, none reported further suicide attempts after initiating the medication [69].

Hassan found no significant difference between clozapine and other antipsychotics in reducing suicidal ideation, suggesting that its protective effect might be more related to behavioral stabilization rather than direct mood improvement [68]. The main disadvantage of that study was small sample size; among 103 participants, 30% experienced suicidal ideation at baseline, and clozapine was used only by 28 individuals (27.2%) [68].

Clozapine significantly reduces suicide risk in patients with schizophrenia and schizoaffective disorder, outperforming other antipsychotics. Its anti-suicidal effect is dose-dependent and diminished by treatment discontinuation. Its benefits may stem from both superior symptom control and increased clinical monitoring due to required safety protocols, a factor also noted with lithium.

Data on the efficacy of clozapine, published between 2014 and 2024, came largely from huge cohort studies conducted in Scandinavian countries with long follow-up. However, the lack of experimental studies (RCTs) makes it difficult to generalize the results more broadly. There are also a small number of studies from other areas of the world and studies with a control group used for comparison. In addition, only one study included analysis of used dosages used and how they might cause the observed anti-suicidal effect.

## 4. Discussion

In this literature review, we looked at scientific studies on suicidal outcomes with the use of lithium, ketamine, and clozapine dating from 2014 to 2014.

Lithium is strongly supported as effective in long-term suicide prevention, especially in bipolar disorder, showing greater efficacy than other mood stabilizers like valproate or lamotrigine. However, discontinuation and poor adherence—ranging from 76.5% to as low as 17%—remain major concerns, linked to both treatment demands and disease characteristics [38,41]. Regular monitoring may help reduce suicide risk by increasing patient–clinician contact, a factor to consider in future guidelines [33,37]. Research from 2014 to 2024 covered diverse populations across regions and diagnoses. Large registry-based cohort studies included thousands, while RCTs had much smaller samples, only two with around 500 participants and two with under 100, limiting generalizability due to high heterogeneity.

Ketamine shows rapid but short-lived anti-suicidal effects, often reducing suicidal thoughts within hours or days—faster than any other known drug. This makes it promising for acute suicidal crises, especially since no drug has been proven to reduce suicidal behavior in depression. Unlike antidepressants, which take weeks to work and lack anti-suicidal efficacy, ketamine acts quickly. However, its effects fade over time, requiring repeated use or combination therapies. Evidence on long-term benefits is mixed, and large real-world studies are still needed before it can be widely recommended. Most ketamine studies used a consistent dose (0.5 mg/kg IV), aiding comparison, though some variation existed. The majority were RCTs, theoretically reducing bias, but sample sizes were small, usually under 100 participants, limiting their strength.

Clozapine has been consistently associated with reduced suicide risk in patients with schizophrenia and schizoaffective disorder. Cohort studies indicate that its use significantly lowers both suicide attempts and completed suicides compared to other antipsychotics. However, discontinuation of clozapine increases suicide risk, similar to lithium. The anti-suicidal effects appear to be dose-dependent, further reinforcing the importance of sustained treatment adherence. Additionally, the stringent monitoring required due to the risk of clozapine-induced blood disorders ensures more frequent clinical follow-ups. This increased medical oversight allows for earlier detection and intervention in cases of suicidality, potentially contributing to the reduced suicide rates observed in clozapine-treated patients. A similar protective effect has been suggested for lithium, where regular monitoring may facilitate timely management of suicidal risk [74]. Most data on clozapine’s efficacy come from large, long-term Scandinavian cohort studies. However, the lack of RCTs and limited studies from other regions or with control groups limit broader applicability. Only one study analyzed how dosage may influence its anti-suicidal effects.

### 4.1. Limitations

Despite promising evidence, several limitations remain. There are still question marks over how well the three aforementioned medications can work in the general population of real-world clinical settings. First, most of the data come primarily from observational studies. Only a few large experimental studies have been conducted. Ketamine has the highest number of RCTs, but they were conducted on fairly small groups. The specific location of a significant number of studies (studies on lithium conducted mainly in the U.S., studies on clozapine mainly in Scandinavian countries) also makes it difficult to extrapolate results to other populations. Perhaps the results are not so replicable in completely different healthcare systems and national suicide prevention strategies. Likewise, there is a lack of studies from middle- and/or low-income countries.

Also, in studies based on large registries of patients with long follow-up, variability of diagnoses and instability over time can be a problem. In some studies, recruited patients were diagnosed on the basis of the International Classification of Diseases 9th revision (ICD-9) and then ICD 10; also, DSM revisions have changed quite significantly over the years.

In addition, heterogeneity in outcome measurements also plays a significant role. Studies use various scales and/or different endpoints (suicide deaths, suicide attempt, hospitalization after suicide attempt), making cross-trial comparisons challenging. Safety and tolerability concerns also play a crucial role in treatment feasibility, with clozapine’s significant side-effect profile limiting its broader use, the need for close monitoring of lithium levels at the beginning of treatment, and ketamine necessitating stringent monitoring. Real-world adherence and tolerability may differ from controlled trial conditions, impacting the effectiveness of these treatments outside research settings. Additionally, mechanistic uncertainties persist, as the precise biological pathways through which these medications reduce suicidality remain difficult to isolate in clinical populations with complex psychiatric conditions.

All of these issues make it difficult to generalize the conclusions of these studies and make it more difficult to establish an accurate cause-and-effect mechanism. Without large longitudinal and/or randomized studies, we will not be able to answer questions about causality.

### 4.2. Future Directions

Future research directions on pharmacological prevention of suicide should include larger-scale, multi-center RCTs examining the long-term outcomes. However easy it is to say so, under practical conditions the organization of such a study can be significantly hampered.

First, death by suicide even in countries with the highest number of them averages about 15–20 deaths per 100,000 population per year [1]. This means that assembling a large enough general population cohort that can be followed for a reliable period of time may require recruiting hundreds of thousands or millions of participants. One can try to circumvent this obstacle by focusing on selected high-risk groups.

However, another major challenge is the paradigm shift in recruiting patients for clinical trials of psychiatric drugs. To date, a significant number of trials of antidepressants and antipsychotics exclude patients at increased risk of suicide [75]. Excluding patients at high risk of suicide may limit the comprehensiveness and generalizability of the findings. However, this approach raises important ethical considerations, as certain pharmacological treatments may in some circumstances elevate suicide risk—a factor that is not always identifiable at the initiation of therapy [76].

It is also necessary to study new drugs or those that have shown unexpected anti-suicidal effects in past studies, such as aripiprazole and others [77]. Also, many types of psychotherapy or electroconvulsive therapy have shown anti-suicidal effects in studies and thus are worthy of further investigation, including in clinical trials of combining psychiatric drugs with different treatment modalities [4,75].

In many developed regions, suicide in the pediatric population (<18 years old) is emerging as a growing problem, becoming one of the leading causes of death in this age population [1]. Unfortunately, we have very scant data on suicide prevention in this age group. Most of the studies analyzed in this article included only adult patients.

Finally, it may not be fully possible to enforce suicide prevention as simply just a drug treatment. Suicide is a complex phenomenon involving both numerous individual and broader social and economic variables [75]. Comprehensive suicide prevention in the general population and specific risk groups should also be based on controlling access to methods, school programs, and ensuring access to mental health care [2].

## 5. Conclusions

While lithium, ketamine, and clozapine each show varying degrees of reducing suicidal behavior, their effectiveness seems influenced by adherence, patient characteristics, and health system factors. Lithium for BD and clozapine for schizophrenia offer more robust long-term protective effects, while ketamine provides rapid but transient relief, particularly useful in acute settings. However, the current evidence base, largely reliant on observational studies and limited by methodological inconsistencies, highlights the need for larger, well-designed trials to guide clinical practice and inform future treatment guidelines.

## Figures and Tables

**Table 1:** Studies on lithium and suicidality.

Reference Number	Main Author	Study Design	Sample Size	Lithium (Serum Level)	Control (Dose)	Inclusion Criteria	Exclusion Criteria	Main Findings
[30]	Kanita Dervic 2023	2.5 year randomized, double-blind trial	94	0.6–1.0 [mEq/dL]	valproate; 45–125 µg/mL serum level	BD patients (DSM-IV) with at least one past suicide attempt; in depressive or mixed episode; aged 18–75 years	lack of capacity to provide informed consent; pregnancy or lactation; active medical problems, including substance abuse problems requiring detoxification; contraindication to use of lithium or valproate; a history of nonresponse to adequate dosages of either lithium and valproate in the past 2 years; contraindication to the use of adjunctive antidepressants if in a depressed state or adjunctive antipsychotics if in a mixed or psychotic depressed state.	significant antisuicidal effect of lihium in BD patients > 42 yo, independent of clinicals and sociodemographic characteristics, effect not observed for younger patients group
[31]	Francesca Girlanda 2014	underpowered RCT	58	0.57 [mEq/dL] (mean)	usual care	diagnosis of unipolar major depression (DSM-IV); an episode of Deliberate Self-Harm in the previous 12 months; inadequate response to at least two antidepressants given for the current depressive episode; uncertainty about which treatment arm would be best for the participant; age 18 or above; informed consent	a primary diagnosis of any concurrent Axis I disorder (DSM-IV) other than major depression; previous exposure to lithium associated with lack of efficacy or adverse reactions; clinical conditions contraindicating lithium; pregnant/lactating women and women of childbearing potential not practicing a reliable method of contraception.	study failed to achieve, minimum sample size needed to detect clinically meaningful difference, source for further meta-analyses
[32]	Szmulewicz A. 2023	double-blind, placabo controlled, randomized trial	486	0.6–0.8 [mEq/dL]	placebo	U.S. Veterans with a diagnosis of major depressive disorder or bipolar disorder with a suicidal event in the prior 6 months	clinical conditions that justify stopping the study drug; use of medications that justify stopping the study drug; frequency of measurement of lithium levels both during the titration phase and after the titration phase; minimum number of pills of the study drug that participants must use if they had no justified reason to stop treatment; maximum time without a clinical visit.	protective effect of lithium cannot be ruled out, need for further studies
[33]	Kelsie M. Stark 2022	retrospective review	98	0.6–1.2 [mEq/dL]	no control–tracking suicidal behavior in time	U.S. Veterans; aged ≥18 years; had an active lithium prescription on the date of data extraction; had an active lithium prescription for at least 6 months	had <3 months of data before and/or after lithium was used for 6 months; if they were initiated on lithium outside particular healthcare institution	significant antisuicidal effect, but point to a need of prospective placebo-controlled studies due to limitations (sample size, unknown serum levels, etc.), study should be considered as hypothesis-generating
[34]	Ole Brus 2019	register-based cohort study	7350	not measured	standard care	all patients in the Swedish National Quality Register for ECT (electroconvulsive therapy) who had received index ECT for unipolar depression between 2011 and 2016, and were registered as in-patients in the Swedish National Patient Register at the start of the treatment series.	age under 18 years of age; readmission on the same day as discharge; death registered before or on the day of discharge; incomplete data on marital status.	Patients treated with lithium after ECT for unipolar depression were less likely to die by suicide or be readmitted
[35]	Elena Toffol 2015	nationwide registry-based prospective cohort study	826	not measured	standard care	all the individuals who were hospitalized in Finland because of a suicide attempt between 1 January 1996 and 31 December 2003, and in prospective screening had been hospitalized due to bipolar disorder before the index attempt		lithium non-significantly lowers risk of suicide attempt, is assosiated with decreased suicide mortality and all-cause mortality (by 49%), recommendationf for lithium usage in suicidal BP patients
[36]	Ira R. Katz 2022	randomized clinical trial	519	0.6–0.8 [mEq/dL]	placebo + usual care	U.S. Veterans with a diagnosis of major depressive disorder or bipolar disorder with a suicidal event in the prior 6 months	schizophrenia; 6 or more previous lifetime suicide attempts; use of lithium within the past 6 months; history of significant adverse effects of lithium; unstable substance use or medical conditions; pregnancy, lactation, or not using birth control; participating in another randomized intervention trial; current use of clozapine, haloperidol, or diuretics except amiloride.	adding lithium to existing treatment is unlikely to be effective for preventing a broad range of suicide-related events
[37]	Pao-Huan Chen 2023	nationwide cohort study	25,787	not measured	valproate, lamotrigine, carbamazepine	all from patients with BD diagnosis from Taiwan’s insurance registry (1 February 2001–31 December 2016)	at least one psychiatric diagnosis of schizophrenia	longer duration of lithium use and higher cumulative doses are associated with lower risk of all-cause mortality (both suicidal and natural). much greater then other medications. evidence-based support for lithium as the treatment of choice for patients with bipolar disorder at high risk of suicide
[38]	Eric G Smith 2015	cohort study	93,162	not measured	valproate	U.S. Veterans; at least one psychotic or depresive disorder; incident users receiving at least one out-patient prescription for lithium or valproate from April 1999 to December 2008; broad cohort of patients with mood or psychotic diagnoses in the 30 days prior to medication initiation was examined since the limited prior literature concerning lithium and mortality is not restricted to bipolar disorder	patients with possible non-psychiatric indications for valproate or lithium; initiated lithium or valproate on an ‘as needed’ basis or both medications simultaneously, or resided outside the USA.	lithium lowered suicide rates in general, but in intent-to-treat method effect is visible 0–90 days but not later, majorly due to disontinuation and connected increased risk (as shown in as-treated aproach)
[39]	Joe Kwun Nam Chan 2024	cohort study	8137	not measured	valproate, quetiapine, risperidone, olanzapine	all individuals aged ≥15 years who received a first-recorded diagnosis of BD for public psychiatric inpatient or outpatient treatment in Hong Kong between 1 January 2002 and 31 December 2018.	individuals with their diagnosis changed to schizophrenia or schizoaffective disorder; BD patients who had not been exposed to any of the five studied mood stabilizers	supports usage of lithium as first-line mood stabilizer for BD. lithium, quetiapine and valproate are associated with lower risk of all-cause death compared to risperidone and olanzapine. doesnt differenciate risks betwen lithium, quatiepine and valproate.
[40]	Jie Song 2017	prospective study	51,535	not measured	valproate and without mood stabilizer	BD patients followed from 1 October 2005, or age 15, or date of first diagnosis if later than 1 October 2005, until emigration, death, or 31 December 2013, whichever occurred first; data acquired from drifferent Swedish National Registries		lithium decreased suicide rate by 14% compared to valproate. lithium should by considerd for patients with BD with suicidal intentions. estimation that more than 10% of all suicide-related events could be prevented if all patients had been treated with lithium. points to risk of discontinuation
[41]	Eric G Smith 2022	cohort study	23,298	not measured	valproate	Veterans Health Administration (VHA) patients with mental health diagnoses initiating lithium or valproate from April 1999 to December 2008; all individuals with recent VHA utilization were identified among all individuals with a mood or psychotic diagnosis in the past 30 days who received at least one outpatient prescription of lithium or valproate; incident recipients within this cohort were identified as individuals with ≥6 months of no lithium or valproate use prior to initiation	individuals with potentially nonpsychiatric indications for lithium or valproate treatment; individuals starting both lithium and valproate simultaneously	decreased one-year suicide risk, but also increased (14/20-fold) suicidal behaviour after discontinouation, decreased suicide risk for not bipolar patients.
[42]	Eric G Smith 2014	historical prospective cohort study	93,335	not measured	valproate	U.S. Veterans; at least one psychotic or depresive disorder; incident users receiving at least one out-patient prescription for lithium or valproate from April 1999 to December 2008; broad cohort of patients with mood or psychotic diagnoses in the 30 days prior to medication initiation was examined since the limited prior literature concerning lithium and mortality is not restricted to bipolar disorder	patients with possible non-psychiatric indications for valproate or lithium; initiated lithium or valproate on an ‘as needed’ basis or both medications simultaneously, or resided outside the USA.	no significant change in suicidal behaviour on lithium, but increased risk of suicide death after discountinuation of lithium

BD—bipolar disorder; USA—United States of America; ECT—electroconvulsive therapy; ICD—International Classification of Diseases; DSM—Diagnostic and Statistical Manual of Mental Disorders; VHA—Veterans Health Administration.

**Table 2:** Studies on ketamine and suicidality.

Reference Number	Main Author	Study Design	Sample Size	Ketamine (Dose)	Control (Dose)	Inclusion Criteria	Exclusion Criteria	SI-Scale	Main Findings
[43]	Grunebaum et al., 2017	Double-blind randomised trial	N = 16 K = 7 C = 9	0.5 mg/kg infusion + current psychotropics except for benzodiazepines	Midazolam 0.02 mg/kg + current psychotropics	Age 18–65 DSM IV BD and MDE scoring HDRS-17 ≥ 16 and SSI ≥ 4	unstable medical or neurological illness, significant (ECG) abnormality, pregnancy or lactation, current psychosis, history of ketamine abuse or dependence, other drug or alcohol dependence within 6 months, suicidality due to binge substance use or withdrawal, prior ineffective trial of or adverse reaction to ketamine or midazolam, daily opiate use > 20 mg oxycodone or equivalent during the 3 days pre-infusion, score < 25 on the Mini Mental State Exam18 (for subjects > 60 years old)	SSI assessed at screening, baseline (day before infusion), 230 min post-infusion, day 1, and weeks 1–6 of follow-up.	Mean reduction of SSI 24 h after ketamine infusion was almost 6 points greater than after midazolam, although this was not statistically significant.
[44]	Fineberg et al., 2023	RCT	N = 22 K = 10 M = 12	0.5 mg/kg	Midazolam 0.04 mg/kg	21–60 yrs current suicidal ideation borderline personality disorder	Current acute risk of suicide Psychotic disorder Substance abuse Current intake of topiramate, lithium, lamotrigine Exposure to ketamine in the past year	BSS	Ketamine did not lead to a significant decrease in suicidal ideation compared to midazolam
[45]	Phillips et al., 2020	Double-blind randomised cross-over comparison	N = 37	0.5 mg/kg	Midazolam 0.03 mg/kg	Age 18–65 MDD DSM-IV TRD MADRS ≥ 25 Item 10 MADRS-SI ≥ 2	history of substance use disorder, body mass index ≥ 35 and unstable medical conditions.	MADRS-SI	Greater reduction of SI 2 h and 7 days post infusion, with greatest effect at 7 days. SI scored did not differ at 24 h Scores were not assessed 24 h–7 days.
[46]	Abbar et al., 2022	Double-blind randomised placebo controlled trial	N = 156 K = 73 C = 83	Two 0.5 mg/kg infusions, 24 h apart	Two placebo 0.9% saline infusions 24 h apart	Age ≥ 18 SSI > 3	Psychotic disorders schizoid or schizotypic personality disorders; presence of psychotic symptoms at initial interview; substance dependence; positive urine screening for illicit substances; pregnancy or breastfeeding; unstable somatic condition; known or suspected contraindication for ketamine; clinically important anomalies found during clinical examination, biological tests or electrocardiogram; non-stabilised hypertension or hypertension > 180/100; concomitant electroconvulsive therapy; current participation or participation within the past three months in another interventional study; patients under judicial protection or guardianship.	Clinician rated SSI score Full suicidal remission assessed as SSI < 3	63.0% reached full remission) three days after two infusions in the ketamine group in comparison with 31.6% in the placebo group. This effect was rapid, with 43.8% remission only two hours after the first infusion versus 7.3% in the placebo group. Until week 6, the ketamine arm continued to have better full suicidal remission than the placebo arm although this was not significant at week 6 owing to reduced suicidality in the placebo group over time
[47]	Canuso et al., 2018	Double-blind randomised placebo controlled study	N = 86 K = 36 C = 32	Esketamine 84 mg twice weekly for 4 weeks + standard of care medication	Placebo Twice weekly for 4 weeks + standard of care medication	Age 19–64 MDD-DSM IV without psychotic features Affirmative response to MINI question B5 and B9 MADRS ≥ 22	current diagnosis of bipolar disorder, moderate to severe substance use disorder, intellectual disability, antisocial personality disorder, borderline personality disorder, or a current or past diagnosis of a psychotic disorder.	MADRS-SI Clinician global judgement of suicide risk	Esketamine group had significantly greater improvement in score on the MADRS suicidal thoughts item 4 h after first dose but not at 24 h or at double-blind end point-day 25. Analysis of the clinician global judgment of suicide risk showed numerically greater, but not statistically significant, decreases in ratings 4 and 24 h after the first dose.
[48]	Grunebaum et al., 2017	RCT	N = 80 K = 40 C = 40	0.5 mg/kg + current psychiatric medication except benzodiazepines	Midazolam 0.02 mg/kg + current psychiatric medication except benzodiazepines	18–65 yrs MDD HDRS-17 ≥ 16 and SSI ≥ 4	unstable medical or neurological illness, significant ECG abnormality, pregnancy or lactation, current psychosis, history of ketamine abuse or dependence, other drug or alcohol dependence within six months, suicidal ideation due to binge substance use or withdrawal, prior ineffective trial of or adverse reaction to ketamine or midazolam, daily opiate use greater than 20 mg oxycodone or equivalent during the three days pre-infusion, score < 25 on the Mini Mental State Exam (25) for persons > 60 years old, lack of capacity to consent or inadequate understanding of English.	Clinician rated SSI	A single ketamine infusion was associated with greater reduction in suicidal thoughts at 230 min and 24 h post infusion, compared to midazolam control
[49]	Fu et al., 2020	Double-blind placebo controlled randomised study	N = 226 K = 114 C = 112	84 mg esketamine nasal spray twice weekly for 4 weeks + standard of care	Placebo spray twice weekly for 4 weeks + standard of care	18–64 MDD-DSM V with MADRS > 28 and in need of acute psychiatric hospitalization due to imminent suicide risk	bipolar disorder, obsessive-compulsive disorder, antisocial personality disorder, borderline personality disorder, substance or alcohol use disorder within 6 months prior to screening, diagnosis of psychotic disorder	SIBAT	At the 24-h endpoint, patients in both treatment groups experienced improvement in the severity of their suicidality as measured by CGI-SS-r, though there was no statistically significant difference between treatment groups Improvement in severity of suicidality was also observed in both treatment groups at the end of double-blind treatment.
[50]	Zolghadriha et al., 2024	RCT	N = 64 K = 32 C = 32	0.5 mg/kg	Saline	25–60 yrs old MDD-DSM 5 MARDS > 25 TRD	uncontrolled hypertension, active substance abuse, or a history of psychosis	BSI	Decrease in suicidal ideation was observed specifically in the group that received ketamine treatment
[51]	Ionescu et al., 2020	RCT	N = 227 K = 114 C = 113	84 mg of esketamine twice weekly for four weeks+ standard of care	Placebo + standard of care	18–64 MDD-DSM V MADRS > 28 Suicidal ideation with intent	bipolar disorder, obsessive-compulsive disorder, antisocial personality disorder, borderline personality disorder, substance or alcohol use disorder within 6 months prior to screening, diagnosis of psychotic disorder	SIBAT	Patients in both treatment groups experienced rapid reduction in the severity of their suicidality, the between-group difference was not statistically significant
[5]	Ahmed et al., 2023	RCT	N = 36 K = 18 C = 18	Two infusions of 0.5 mg/kg ketamine one week apart	Two infusions of saline one week apart	18–65 TRD MDD-EMA Current suicidal risk	major unstable medical condition or neurological illness, the presence of perceptual disturbance, a history of sensitivity to ketamine, a history or present substance use, and an ECT treatment in the previous 3 weeks	Suicide probability scale	Significant reduction in SPS in ketamine group, no change in placebo group 1 week after finishing 2 infusions.
[52]	Sinyor et al., 2018	Randomised controlled trial	N = 9 K = 5 C = 4	0.5 mg/kg 6 infusions over 12 days Adjunct to treatment	Midazolam 0.045 mg/kg 6 infusions over 12 days	18–65 MDD SSI or CSSRS > 0 MDD did not have to be treatment resistant No minimum required depression score	(1) current or past manic symptoms; (2) current or past psychotic symptoms; (3) current substance or alcohol dependence or substance abuse within the past month; (4) pervasive developmental disorder or dementia; (5) unstable medical illness; (6) medical condition that would contraindicate the use of ketamine/midazolam or affect their metabolism; (7) current treatment with ketamine; (8) any treatment that might contraindicate the use of study medications (9) concomitant electroconvulsive therapy; and (10) pregnancy.	SSI	Subjects in both the ketamine and midazolam groups experienced a rapid reduction in SI, the reduction in the ketamine group appeared to be somewhat larger, more robust, and longer-lasting (up to 42 days) with SSI scores increasing in the midazolam group after the second infusion.
[53]	TP Su 2023	RCT	N = 84 K = 42 C = 42	0.5 mg/kg Single infusion	0.045 mg/kg Midazolam	20–64 MDD-DSM V TRD MADRS item 10 ≥ 4	major medical or neurological diseases or a history of alcohol or substance use disorders in current study	CSSRS-ISS MADRS item 10 PANSI	More patients receiving ketamine reached full remission of suicidal ideation from day 2 to day 5 compared with those receiving midazolam. Significant antisuicidal effects of low-dose ketamine infusion at least up to day 5.
[54]	Price 2014	RCT	N = 57 K = 36 C = 21	0.5 mg/kg Single infusion	Midazolam 0.045 mg/kg	MDD TRD Moderate-severe depression Psychotropic medication free	Serious and imminent suicidal or homicidal risk Psychotic disorder Bipolar disorder Substance abuse	Explicit SI composite score (BSS, MARDS-S, QIDS-SI)	Tweny-four hours postinfusion, ketamine-treated patients exhibited large, rapid reductions in explicit suicidal cognition, which were significantly greater than reductions observed in midazolam-treated patients. Ketamine eradicated all self-report and clinician-rated indications of suicidal ideation in 53% of patients, compared to 24% of patients receiving midazolam
[55]	Fan et al., 2016	RCT	N = 39 K = 20 C = 19	0.5 mg/kg	Midazolam 0.05 mg/kg	18–70 First diagnosed with cancer 3 months	cardiorespiratory diseases; drug addiction history or sedative–hypnotic drug(s) use; neuropsychiatric or cognitive diseases or a related treatment history; suicidal attempts or ideation before cancer diagnosis; and family history of psychiatric history.	BSS MARDS-SI	Suicidal ideation significantly lower in ketamine group at day 1 and day 3 post infusion
[56]	Ionescu et al., 2019	RCT	N = 26 K = 13 C = 13	0.5 mg/kg Six infusions over three weeks	Saline Six infusions over three weeks	18–65 MDD-DSM IV HDRS ≥ 20 C-SSRS > 1 for 3 months + HDRSi12 ≥ 4	(1) pregnancy; (2) unstable medical illness; (3) bipolar disorder; (4) past multiple adverse drug reactions; (5) psychotic illness; (6) substance use disorder within the past year; (7) positive urine toxicology; (8) past history of ketamine abuse; (9) SI requiring immediate hospitalization or indicating immediate	C-SSRS	Ketamine treatment did not have a significant advantage in terms of reducing SI.
[57]	Murrough et al., 2015	RCT	N = 24 K = 12 C = 12	0.5 mg/kg Single infusion	Midazolam 0.045 mg/kg Single infusion	18–80 MADRS-SI ≥ 4 Range of psychiatric diagnoses	C-SSRS 4 or 5 Schizophrenia or other primary psychotic disorder, current psychotic or manic symptoms, substance use disorder within 1 month of screening,	BSS MADRS-SI	BSS score was not significantly different between the treatment groups 24 h post treatment A significant effect of treatment on BSI score emerged at 48 h following intervention. 24 h following treatment, MADRS-SI score was significantly lower in the ketamine group compared to midazolam group
[58]	Feeney et al., 2021	RCT	N = 56 K = 40 C = 16	0.1 mg/kg 0.5 mg/kg 1.0 mg/kg	Midazolam 0.045 mg/kg	18–70 MDD of at least 8 weeks prior to screening TRD MADRS > 20 MADRS-SI ≥ 2	Imminent risk of suicide More than seven adequate antidepressant trials in current episode	MADRS-SI	Those who had received IV ketamine had a significant reduction in suicidal ideation compared to those who had received IV midazolam. However, when participants with an apparent early anti-suicidal response to the infusion were analyzed, treatment and placebo groups did not differ significantly at days 5, 7, 14 or 30 post infusion.
[59]	Domany et al., 2020	RCT	N = 18 K = 9 C = 9	0.2 mg/kg over 5 min Single infusion	Saline Single infusion	18–65 MDD Clinically significant suicidal ideation	Psychotic disorder Anorexia nervosa Substance abuse Pregnancy, post-partum	BSS MARDS-SI	A reduction in suicidal ideation was noted at 90–180 min Ninety minutes after infusion, 88% of the ketamine group had achieved remission of suicidal ideation compared with 33% in the placebo group
[60]	Zeng et al., 2025	RCT	N = 40 K = 20 C = 20	Esketamine 0.5 mg/kg + ECT	Propofol 1.5–2.5 mg/kg + ECT	18 and 65 years TRD MDD (DSM-IV) HAMD-17 scores ≥ 17.	(1) Other psychiatric disorders, However, individuals with comorbid anxiety disorder, obsessive-compulsive disorder, or eating disorder were allowed provided that these conditions were not the primary presenting issue; (2) severe physical disease; (3) pregnancy, planning to get pregnant, or breastfeeding; (4) having received ECT or repetitive transcranial magnetic stimulation within the previous three months; and (5) having allergies to the anesthetic agents.	SSI-part1	Antisuicidal effects in SSI-part 1 scores did not differ significantly between the two groups
[61]	Hu et al., 2016	RCT	N = 30	Four weeks escitalopram 10 mg/day + single infusion 0.5 mg/kg ketamine	Four weeks escitalopram 10 mg/day + single saline infusion	Outpatients with non-psychotic MDD HAMD ≥ 24 ≥1 item 3 HAMD 18–60	Psychotic, bipolar, OCD disorders Substance abuse Suicide attempt in the current episode	QIDRS-SR item 12	Escitalopram+ketamine was associated with significantly lower QIDRS-SR suicidality item scores from 2 to 72 h
[62]	Burger et al., 2016	RCT	N = 10 K = 3 C = 7	0.2 mg/kg single ketamine infusion over 2 min	Saline over 2 min	Patients presenting to military ED with depression and suicidal thinking BSS > 4 BHS > 8	Psychotic disorder Bipolar disorder Substance abuse	BSS	67% of subjects who received ketamine presented significant improvement in suicidal ideation in the emergency department (4 h)
[63]	Jones et al., 2024	RCT	N = 28 K = 17 C = 11	50 mg IN ketamine	Saline	21–60 Current SI Past suicide attempt MDD	Psychotic disorders Substance abuse-excluding alcohol	SSI MARDS-SI	No significant improvement was observed in SSI Significant response observed in MARDS-SI
[59]	Domany et al., 2021	RCT	N = 30 K = 15 C = 15	40 mg IN ketamine	Saline	Required psychiatric hospitalisation due to suicidal risk	Schizophrenia spectrum disorders Dissociative disorders Cognitive disorders Substance abuse	BSS MARDS-SI	A significant reduction in suicidal ideation was observed 4 h after administration 80% achieved suicidal remission compared to 33% in placebo group

N—number; K—ketamine; C—control; DSM-IV—Diagnostic and Statistical Manual of Mental Disorders; BD—bipolar disorder; MDE—major depressive episode; HDRS-17—17 item Hamilton Depression Rating Scale; SSI—scale for suicidal ideation; ECG—electrocardiogram; BSS—beck scale for suicidal ideation; MDD—major depressive disorder; TRD—treatment resistant depression; MARDS-SI—suicide item Montgomery-Åsberg Depression Rating Scale; SI—suicidal ideation; SSI—scale for suicidal ideation; MINI—Mini International Neuropsychiatric Interview; SIBAT—Suicide Ideation And Behaviour Assessment Tool; CGI-SS-r—Clinical Global Impression of Severity of Suicidality Revised version; SPS—Suicide Probability Scale; EMA—European Medicines Agency; CSSRS-ISS—Columbia Suicide Severity Rating Scale-Ideation Severity Subscale; PANSI—Positive and Negative Suicide Ideation Inventory; QIDS-SI—Quick Inventory of Depressive Symptomatology-Suicide Item; ECT—Electroconvulsive Therapy; OCD—Obsessive-Compulsive Disorder; ED—Emergency Department; BHS—Beck Hopelessness Scale.

**Table 3:** Studies on clozapine and suicidality.

Reference Number	Main Author	Study Design	Sample Size	Control	FOLLOW UP	Inclusion Criteria	Exclusion Criteria	Main Findings
[64]	Gunilla Ringbäck Weitoft 2014	A population-based cohort study (Sweden)	Total sample = (*n* = 26,046) 2138 (8.2%) 13 suicide deaths 42 suicide attempts	Those who did not die specifically from suicide and those who did not have a hospital-stay because of suicide attempt.	For prescription refill analysis, patients were followed for 12 three-month periods (quarters 1, 3, 5, 7, 9, and 11). For re-hospitalisation analysis, the follow-up period started from day 183 after the index care event and lasted until day 365.	Adults (18–79 years old) Patients diagnosed with schizophrenia (ICD-10: F20) or schizoaffective syndromes (F25) Patients who received hospital or specialized outpatient care for schizophrenia in Sweden between 2006 and 2009	Patients who died from other causes before the fictitious date were excluded.	Clozapine users had a lower odds ratio (OR) for death by suicide (OR = 0.45, 95% CI: 0.20–0.98) and for attempted suicide (OR = 0.44, 95% CI: 0.28–0.70) compared to haloperidol users. Clozapine users also had a lower risk of re-hospitalization and higher prescription refill rates. A calculation suggests that using clozapine instead of first-generation antipsychotics could have prevented 95 suicide attempts during the study period.
[65]	van der Zalm 2021	cohort study (Denmark)	The incidence cohort included 22,110 The prevalence cohort included 50,881 Clozapine patients 1677	No control group	The study followed the cohort over a long-term period, taking data from 1995 (when the Danish National Prescription Registry started) up to 2015 (the last available data in the Causes of Death Register).	Patients had to have a diagnosis of schizophrenia or a related psychotic disorder: Patients aged 15 to 100 Patients had to be inhabitants of Denmark with a first diagnosis between 1 January 1995, and 30 June 2013 (incidence cohort). A prevalence cohort included all individuals ever diagnosed with up to 30 June 2013.	Migrants to Denmark were excluded	Current use of clozapine was associated with a lower risk of suicide compared to other antipsychotics. In the prevalence cohort, users of other antipsychotics had a significantly higher risk of suicide compared to clozapine users (HR_adj = 2.20; 95% CI 1.35–3.59). Increased Suicide Risk After Clozapine Discontinuation Cumulative use of clozapine for up to 1 year was associated with a higher risk of suicide compared to other antipsychotics (HR_adj = 0.65; 95% CI 0.46–0.91).
[66]	Taipale 2020	cohort study—(Sweden and Finland)	Finnish cohort: 61,889 individuals with schizophrenia. Swedish cohort: 29,823 individuals with schizophrenia.	No control group	follow-up periods of up to 22 years (Finland) and 11 years (Sweden).	ICD-10 codes (F20, F25) and older equivalents Finland: All individuals treated for schizophrenia in inpatient care from 1972 to 2014. Sweden: All individuals aged 16–64 with a recorded schizophrenia diagnosis between 2006 and 2013. Participants were not institutionalized for long-term care.	Delusional disorder (F22) and nonspecific psychosis (F29) were excluded to maintain a homogeneous cohort. In the incident cohort, individuals were excluded if they had used antipsychotics in the year before their first schizophrenia diagnosis.	Clozapine was the only antipsychotic consistently associated with a decreased risk of suicidal outcomes: Finnish cohort: Hazard Ratio (HR) = 0.64 (95% CI: 0.49–0.84). Swedish cohort: HR = 0.66 (95% CI: 0.43–0.99). No other antipsychotic showed a statistically significant reduction in suicide risk.
[67]	Taipale 2021	register-based cohort study	Finland cohort: 62,250 individuals with schizophrenia)	No control group	Follow-up duration: Up to 20 years (median: 14.1 years	Diagnosis of Schizophrenia Defined using ICD-10 codes F20 (schizophrenia) and F25 (schizoaffective disorder) Older ICD versions (ICD-9 & ICD-8: code 295) were also used to identify cases in earlier years Nationwide Cohort in Finland Included all individuals hospitalized for schizophrenia in Finland between 1972 and 2014	Psychotic disorders other than schizophrenia In the incident cohort (first-episode patients), individuals were excluded if they had used antipsychotics in the year before their first schizophrenia-related hospitalization.	The adjusted hazard ratio (aHR) for suicide mortality was 0.21 (95% CI: 0.15–0.29) for clozapine, indicating a 79% reduction in suicide risk compared to non-use.
[68]	Hassan 2021	Prospective cohort study (Canada)	103 participants	No control group	average follow-up duration of 17.4 ± 7.4 months	Participants were included if they: Were older than 18 years. Had a diagnosis of schizophrenia or schizoaffective disorder. Were fluent in English. Were being treated as outpatients at the Centre for Addiction and Mental Health (CAMH), Toronto.	Participants were excluded if they had psychosis due to traumatic brain injury or a general medical condition and/or were not fluent in English.	The study found that clozapine did not show a significant difference in reducing suicidal ideation compared to other antipsychotics. Here are the exact results: Mean Beck Scale for Suicidal Ideation (BSS) score at follow-up: Clozapine group: 1.93 ± 4.63 Non-clozapine group: 1.67 ± 4.3 Reduction in BSS score: Clozapine group: −0.93 Non-clozapine group: −0.8 *p* = 0.847 (not statistically significant)
[25]	Chen 2024	Cohort study (Taiwan)	Study population = 43,025 Of them, we selected those who received clozapine (clozapine cohort, *n* = 5800).	For each patient in the clozapine cohort, authors selected two individuals from inpatients with schizophrenia who had never used clozapine after matching them by age, sex, and the year of the index date at a ratio of 1:2	The study followed up patients from 1 January 2001, to 31 December 2019 (18 years). The mean follow-up period for the study cohort was 7.9 years.	Population: 43,025 inpatients with schizophrenia from Taiwan’s National Health Insurance Research Database (2001–2019). Clozapine group: 5800 patients. Control group: 11,583 non-clozapine users, matched 1:2 by age, sex, and year of index date. Total cohort: 17,383 patients.	Diagnosis of affective psychosis from 2001 to 2019. Missing information on sex and birth date	Clozapine users had a 63% lower risk of suicide mortality compared to non-users (adjusted hazard ratio (aHR) = 0.37, 95% CI: 0.20–0.67, *p* = 0.001). Dose-dependent effect: Each 10-day increase in clozapine use lowered suicide risk by 26% (aHR = 0.74, *p* = 0.004). Each 10 defined daily doses (DDDs) of clozapine lowered suicide risk by 31% (aHR = 0.69, *p* = 0.010).
[69]	Gürcan 2024	Cross sectional study (Turkey)	122 outpatients with schizophrenia	No control group	No follow up	Single center, diagnosis of schizophrenia (DSM-IV criteria) and who had been receiving clozapine treatment for at least 6 weeks	Not mentioned	Before clozapine: 39.3% of patients had attempted suicide. After clozapine: Only 7.4% attempted suicide (*p* < 0.001). A total of 32% (39 patients) with prior suicide attempts had no further attempts after clozapine.

aHR—Adjusted Hazard Ratio; HR—Hazard Ratio; CI—Confidence Interval; ICD—International Classification of Diseases; OR—Odds ratio; DSM—Diagnostic and Statistical Manual of Mental Disorders.

## Data Availability

Not applicable.
